# En-bloc rectosigmoid and mesorectum resection as part of pelvic cytoreductive surgery in advanced ovarian cancer

**DOI:** 10.4274/jtgga.galenos.2019.2019.0128

**Published:** 2020-09-03

**Authors:** Juan Luis Alcazar, Matias Jurado, Jose Angel Minguez, Enrique Chacon, Fernando Martinez-Regueira

**Affiliations:** 1 Clinic of Obstetrics and Gynecology, Clinica Universidad de Navarra, Pamplona, Spain; 2 Clinic of Surgery, Clinica Universidad de Navarra, Pamplona, Spain

**Keywords:** Ovarian cancer, surgery, rectosigmoid

## Abstract

**Objective:**

“En-bloc” resection of pelvic tumor in ovarian cancer (OC) is still controversial. The aim was to analyze results in an OC series from a single center, all of whom underwent “en-bloc” resection as part of cytoreductive surgery.

**Material and Methods:**

Clinical and surgical records from sixty patients with ovarian carcinoma who underwent “en-bloc” resection surgery were retrospectively analyzed.

**Results:**

Patients’ mean age was 56 years; 36 patients had primary disease and 24 had recurrent disease. Carcinomatosis was present in 46.7% of patients. Primary surgery was performed in 49 and interval debulking surgery in eleven. Complete cytoreduction was achieved in 55.0% and optimal in 38.3% of patients. Carcinomatosis significantly decreased the probability of complete cytoreduction [odds ratio (OR): 0.22; p=0.021]. Mesorectal infiltration occurred in 83% of patients. Risk of death was non-significantly higher (hazard ratio: 1.9) in women with mesorectal infiltration. Median overall survival was longer for patients without infiltration (46.1 vs 79.1 months; p=0.15). Eighty-five percent suffered from mild to moderate complications and colorectal anastomosis (CRA) leak occurred in two patients (3.6%) with CRA below 6 cm. Diaphragm resection had >5 times the risk for major complications (OR: 5.35; p=0.014). There was no three month mortality.

**Conclusion:**

When contiguous gross extension of disease to pelvic peritoneum and sigmoid colon is found, in patients with advanced OC, microscopic involvement of the mesorectum and intestinal wall is present in most cases making “en-bloc” resection necessary if complete cytoreduction is to be achieved. The associated morbidity is acceptable.

## Introduction

Optimal cytoreduction followed by adjuvant chemotherapy with carboplatin and paclitaxel is currently considered as the standard treatment for primary advanced ovarian cancer (AOC) (1,2). To achieve this goal, maximum surgical effort is needed. This may include several intra-peritoneal and extra-peritoneal procedures (3,4,5). The role and potential benefits of a similar surgical therapeutic approach for recurrent OC is still under debate although results from some studies are encouraging (6).

Contiguous extension to pelvic peritoneum and sigmoid colon may need a radical “en-bloc” resection of pelvic tumor with or without colorectal anastomosis (CRA). This procedure has been progressively incorporated into surgical practice and it should be currently considered the standard approach for achieving optimal cytoreduction (7,8,9,10,11,12). Nevertheless, some studies have assessed the real benefit of this more radical procedure balanced against the potential risk of complications (13,14,15,16,17,18,19,20,21,22). It is well known that the spread pattern of ovarian cancer that infiltrates the intestinal wall resembles that of primary colon carcinoma (8,10). This finding would imply that resection of regional lymph nodes of the involved organs, as is undertaken for primary colorectal carcinoma, may be appropriate in optimal debulking surgery (DS) for patients with OC.

The aim of this study was to analyze the results from a series of patients with primary or recurrent OC who underwent “en-bloc” pelvic disease resection with extraperitoneal CRA as part of cytoreductive surgery, in order to investiagte there is oncological justification for this procedure. In addition, the morbidity associated with this surgical approach was documented.

## Material and Methods

Sixty patients with OC, 36 women (60.0%) with a primary tumor and 24 women (40.0%) after recurrence, all of whom underwent “en-bloc” pelvic disease resection with extraperitoneal CRA were retrospectively studied. Clinical and surgical records were retrieved from the hospital database. Institutional Ethical Review Board approval from Clinica Universidad de Navarra (approval number: 2018.001) was obtained for this study. Due to the retrospective design, patients’ informed consent was waived.

The inclusion criterion for this study was any patient with histologic diagnosis of epithelial OC who underwent an “en-bloc” pelvic disease resection with CRA below the Douglas peritoneal reflection.

All patients were followed-up until the time of death or until December 2017, if alive. There was no patient lost to follow-up.

### Surgical procedure

All patients underwent radical oophorectomy according to Bristow et al. (23) technique. The extension of mesorectal resection is partial since the rectum is transected at the same level as the mesorectum, 2 to 3 cm below the palpable tumor and avoiding a cone effect. According to some publications, the oncological outcome of partial mesorectal excision is safe, with a lower risk of anastomotic leak (24).

All CRA were end-to-end and performed with a circular end-to-end anastomotic staple device. Both excised doughnuts were inspected for integrity and macroscopic normality and CRA integrity was checked by a bubble test, and tension and color of the bowel were evaluated. Proximal diversion was not performed. Surgical Complexity Score (SCS) was used for determining the surgical complexity (25).

Preoperatively, most patients received a full mechanical bowel preparation the day before surgery. Standard antibiotic prophylaxis during surgery was delivered. In patients with gross intra-operative fecal contamination, broad-spectrum antibiotic coverage was administered for 72 hours postoperatively.

Low molecular weight heparin at a dose of 3500 U/day was given during the first four weeks and antiembolism stockings and pneumatic sequential compression device were routinely placed during surgery.

### Outcomes analyzed

Primary outcome was oncological outcome. Secondary outcome was associated morbidity.

### Statistical analysis

Statistical analysis was performed with the SPSS for Windows, version 20 (IBM Inc., Chicago, IL., USA). Continuous data are presented as mean with standard deviation or median with interquartile range depending on data distribution. Categorical data are presented as the number of cases and percentages. Categorical data were compared using two-tailed Fisher’s exact test. Kruskal-Wallis test was used where appropriate. Continuous data were compared using Mann-Whitney U test when data distribution was not normal and One-Way analysis of variance when distribution was normal. Odds ratio (OR) with 95% confidence intervals (CIs) for predicting morbidity were calculated for several prognostic factors by using a binary logistic regression analysis, choosing a forward stepping model procedure.

Overall survival (OS) was measured in months from the date of surgery to the time of death or last follow-up appointment. Survival Free of Disease (DFS) was measured from the date of surgery to the time of the first failure observed after surgery. Survival analysis was done with the Kaplan-Meier method, compared by the Log-rank and Breslow statistical method, estimated from the first day after surgery. Univariate Cox proportional hazard ratio (HR) analysis was performed to identify potential prognostic factors and multivariate Cox proportional HR was performed introducing only those statically significant variables found in the univariate analysis. A p value <0.05 was considered statistically significant for all tests.

Sample size calculation was not performed.

## Results

Patients’ demographics, and tumor features are summarized in Table 1. Overall, mean age was 56 years, and patient’s performance was good in 60.0% of cases, Upfront DS was performed in 49 women (82%) and interval DS in eleven (18%). CRA at >6 cm from the anal verge was performed in 96% of patients.

Table 2 shows all surgical procedures performed for achieving maximal cytoreduction. All sixty patients underwent an “en-bloc” pelvic disease resection followed by a CRA below the Douglas reflection. No patient underwent protective fecal stream diversion.

Twenty-eight patients (46.7%) had carcinomatosis, in 12 (20%) patients there were isolated implants and 20 (33.3%) patients had no visible peritoneal disease (p=0.001). Overall, 93.0% of patients had optimal cytoreduction (<1 cm). Complete cytoreduction was achieved in 55.5%. Complete cytoreduction was significantly more frequently achieved in patients with recurrent disease than in those with primary disease (79.2% vs 38.9%; p=0.006). Carcinomatosis was significantly associated with a high SCS (93.0% vs 6.3%; p=0.001) and significantly decreased the probability of complete cytoreduction (OR: 0.22, 95% CI, 0.62-0.80; p=0.02).

### Pathological prognostic factors

Bowel wall infiltration was found in 83.3% (50/60) of cases. The serosa was involved in 22/50 (38.3%) and muscularis-mucosa in 28/50 (56.0%).

The mesorectum was found to be infiltrated by tumor (defined as either directly or through lymph node metastases) in 83% (50/60) of the cases. Mesorectal involvement was associated with the depth of bowel infiltration [36.0% (18/50) if serosa was involved, 56.0% (28/50) if muscularis-mucosa was involved, and 8.0% (4/50) when the bowel wall was not involved]. It should be noted that there were four patients with bowel wall infiltration but not mesorectum infiltration and there were another four patients that had mesorectum infiltration but no bowel wall involvement.

### Survival

After a median follow-up of 37.5 months (95% CI: 38.3-63.7), the 5-year OS and DFS for the whole series were 30.3% and 23.2%, respectively. Median survival time was 47.2 months (95% CI: 34.8-59.5) and median time to recurrence was 18.6 months (95% CI: 12.8-24.5).

The HRs of prognostic factors assessed for OS and DFS are shown in Table 3. Univariate analysis showed a worse outcome for age >65 years, carcinomatosis, residual disease after surgery, high-risk serous tumor, and mesorectum infiltration. In multivariate analysis, only carcinomatosis was significantly associated with OS whereas no residual tumor and age <65 were associated with DFS.

In spite of we did not find mesorectum involvement statistically significant, it should be noted that when the mesorectum was involved the risk of death was 1.9 times higher (HR: 1.9, 95% CI: 0.82-5.2; p=0.157) than when it was not involved. Median OS was 46.1 months (95% CI: 31.9-60.3) and 79.1 (95% CI: 0-172.6) for patients with and without infiltration of the mesorectum, respectively (p=0.15).

All cases with extra-abdominal disease (liver, spleen, suprarenal, chest or others) occurred in patients with mesorectum infiltration. However, despite this pattern of spread, 5-year OS was similar to that of patients with intra-peritoneal and/or retro-peritoneal recurrences (32.0% vs 29.0%; p=0.80).

### Pattern of recurrence

Overall, forty-four (73.3%) patients recurred. Most recurrences were intra-peritoneal (53.3%). It is noticeable that all extra-abdominal (liver, spleen, suprarenal, chest or others) recurrences occurred in patients with mesorectum infiltration. Logistic regression analysis showed that factors related with extra-abdominal recurrence were multiple peritoneal implants [OR: 4.2 (95% CI: 1.02-17.2; p=0.04)] and muscularis-mucosa invasion [OR: 5.4 (95% CI: 1.3-22.4; p<0.01)].

### Morbidity

Five major intraoperative complications occurred: three cystotomies, one vascular injury and one severe hemorrhage. The number of units of blood transfused intra-operativley was higher in the group of patients with recurrent disease (3.2 vs 1.9; p=0.03).

According to the Clavien-Dindo classification (26), most patients suffered from mild to moderate complications (85.0 % of type 1-2, 15% type >3), without statistically significant difference between patients with primary and recurrent disease (p=0.773). CRA leak occurred in two patients (3.6%), both in the group with recurrent disease. One had previous pelvic radiotherapy and the second was undergoing her third adjuvant chemotherapy regimen that included bevazizumab.

Logistic regression analysis showed that pleural effusion, diaphragm resection, infragastric omentectomy, appendectomy, units of blood transfused and surgery length increased the probability of major complications during the postoperative period. In multivariate analysis, diaphragm resection had more than five times the risk of major complications (OR: 5.35, 95% CI: 1.40-20.4; p=0.014), while units of blood transfused postoperatively had a 48% higher risk (OR: 1.48, 95% CI: 0.98-2.23; p=0.06) (Table 4).

There was no mortality during the three month follow-up after surgery.

## Discussion

Rectosigmoid colon with mesorectum resection is considered a standard surgical management for patients with AOC when there is contiguous extension to pelvic peritoneum and sigmoid colon (1). This approach seems reasonable in view of the high frequency of involvement of the distal sigmoid by the ovarian tumor. However, due to the possible associated morbidity there is still reluctance by some to perform this procedure. Histological bowel and mesorectum lymph node infiltration is described in a very large percentage of cases as factors associated with a poorer survival, including this series (7,8,9,10,13,14,15,16,17,18,19,20,21). Some authors suggest that a close colorectal dissection (CRD) without a partial mesorectum excision or adequate longitudinal margin may leave residual tumor in the mesorectum or in the intestinal wall and this is not consistent with the complete disease resection concept (7,8,9,10,13,14,15,16,17,18,19,20,21). A recent study comparing sigmoidectomy with total mesorectal resection (TMR) and without mesorectal resection for removing focal disease did not find difference in progression free survival and concluded that CRD could be an acceptable alternative (27).

It has been suggested that mesorectal infiltration by OC may influence the natural history of the disease through an alternative metastatic pathway similar to the lymphatic or vascular spread of primary intestinal malignancies (7,8,9,10,11,12,13,14,15,16,17). In our series, we observed that liver, spleen, suprarenal and distant extra-abdominal metastasis exclusively occurred in patients with mesorectal infiltration, which is consistent with this concept. Aletti et al. (12) reported that in the subgroup of patients whose pelvic tumor was completely reduced to no visible residual disease by means of a radical proctosigmoidectomy, OS appeared to be superior to those who just underwent pelvic peritonectomy, further reinforcing this hypothesis. Another, similar study did not find the same results and stressed that it was the amount of residual tumor that was independently associated with survival. Therefore, based on this finding and the lower rate of complications without proctosigmoidectomy, systematic mesorectal excision as part OC cytoreduction surgery is not supported (15).

This radical pelvic surgery should contribute to improve survival if disease throughout the abdomen is removed to the point of optimal cytoreduction, as observed in different studies (12,16,18). We observed in univariate analysis that mesorectal infiltration increased the risk of death. However, in multivariate analysis only carcinomatosis influenced this risk. A Mayo Clinic study focused on the prognostic value of FIGO stage IVB due to mucosal colon invasion in patients with no residual disease after surgery and found no correlation of survival with intestinal wall invasion (28). Our results agree with theirs regarding the presence of carcinomatosis as the main prognostic factor for survival and that survival was not associated with mesorectal involvement or depth of colon wall infiltration.

In our series, the risk of death was 90% higher for patients with any amount of residual disease but it only showed a trend toward statistical significance. This could be explained by the small number of patients and the heterogeneity of the group with recurrent disease. Nevertheless, the risk of recurrence was significantly influenced by residual disease.

The pattern of recurrence after this type of surgery for AOC is not well known. In our series, the most frequent recurrence site was the peritoneum followed by retro-peritoneal lymph nodes and similar findings were reported by Amate et al. (29). Some publications focused on pelvic recurrence as the paradigm to explain the benefit of pelvic disease resection with “en-bloc” rectosigmoidectomy and report a lower rate of pelvic recurrence (7,8,10,18,22). In addition, Scarabelli et al. (7) called attention to distant metastases, specifically hepatic metastases. Distant metastasis was more frequent among patients with deep infiltration of the muscularis of the rectosigmoid, mesorectal lymph node involvement and residual tumor >1 cm.

The analysis of risk factors for the spread pattern in our series is similar to those reported by Scarabelli et al. (7), which are volume of disease (multiple peritoneal implants) and deep infiltration of the bowel being the factors associated with the spread of the disease.

Several recent studies dealing with this surgical approach to AOC conclude that the morbidity and mortality following this procedure is acceptable. We reviewed published series that included at least 50 patients who had undergone a colorectal resection with CRA and in which morbidity had been analyzed (7,8,9,10,11,12,13,14,15,16,17,18,19,20). CRA leak occurred in 3.6% (range, 0% to 9.0%), proximal fecal stream diversion was performed in 18.8% (range, 0% to 58.4%), major complications occurred in 23.3% and death in 1% (range, 0-6%) of the cases, respectively. Recently several publications have focused on this topic (27,30). The series from Korea (27) compares the rate of CRA leakage according to whether a TMR or a CRD were performed and they showed a higher rate for TMR (5.3 vs 0%). Multivariate analysis showed postoperative hemoglobin as an independent prognostic factor. Lago et al. (30) in a multinational European centers study of 695 patients found a rate of CRA leakage of 6.6% (1.7-12.5%) despite 37% of them having undergone a diverting ileostomy. Multivariate analysis showed several risk factors for anastomotic leak including manual anastomosis and distance of the anastomosis from the anal verge. Results regarding morbidity in our series are similar to the above-mentioned literature review. We had no deaths within the 90 days following surgery.

### Study Limitations

The main strength of our study is that the pathological examination and reporting allowed us to analyze the relationship between the depth of infiltration of the rectal wall and/or involvement of the mesorectum and the pattern of recurrence. Whether or not extra-abdominal recurrences impact survival would require analysis of a larger group of patients.

Our study has some weaknesses such as the small number of patients, the heterogeneity of the group of patients with recurrent disease, the lack of specificity with regard to the mesorectal involvement being due to nodal metastasis or infiltration of the mesentery, and the lack of a control group. These weaknesses preclude us from drawing strong conclusions.

## Conclusion

Mesorectal and intestinal wall involvement by tumor is frequent in patients with AOC that grossly appears to involve the sigmoid colon, suggesting that performing an “en-bloc” pelvic disease resection may benefit some patients. However, complete or optimal cytoreduction is the main prognostic factor associated with survival. Our data and other authors’ findings suggest that the morbidity and mortality associated with “en-bloc” colorectal resection and anastomosis is acceptable.

## Figures and Tables

**Table 1:**
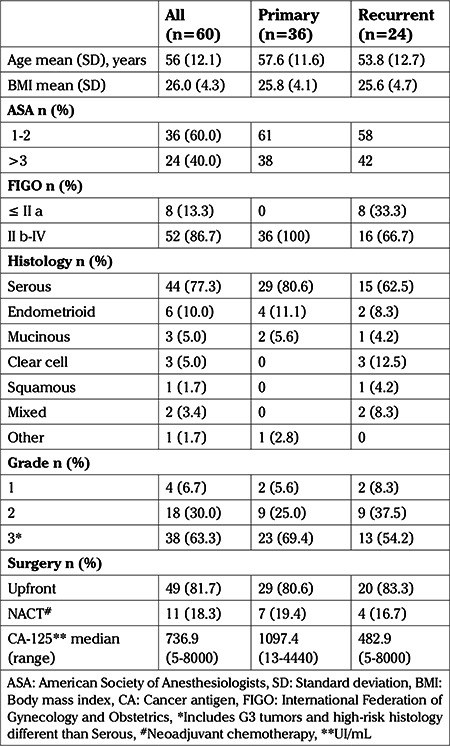
Patients’ and tumor characteristics

**Table 2:**
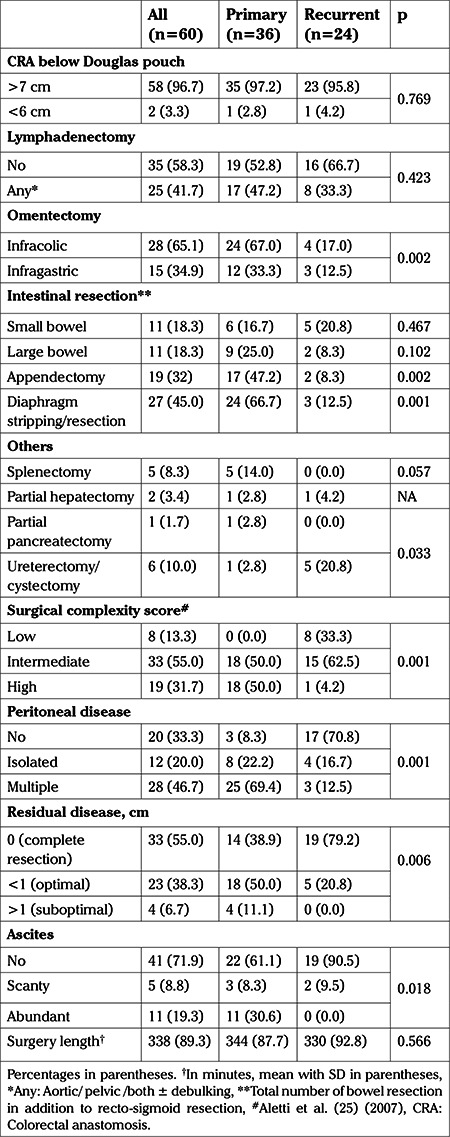
Surgical characteristics of the cases

**Table 3:**
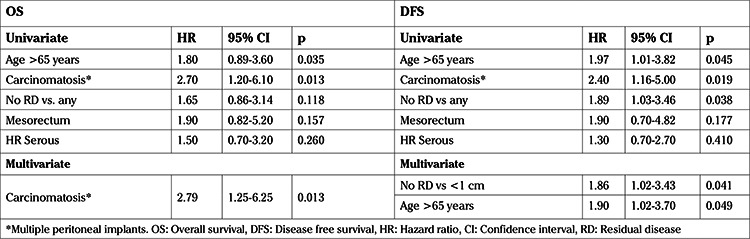
Logistic analysis of co-variates related to survival (Cox regression)

**Table 4:**
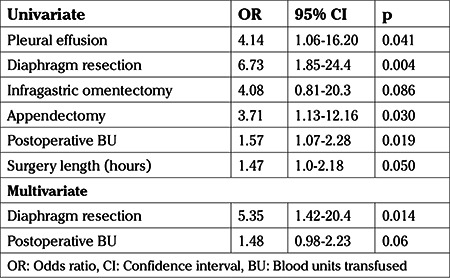
Factors associated with morbidity with Clavien-Dindo score ≥3
